# Triparental plants provide direct evidence for polyspermy induced polyploidy

**DOI:** 10.1038/s41467-017-01044-y

**Published:** 2017-10-18

**Authors:** Thomas Nakel, Dawit G. Tekleyohans, Yanbo Mao, Golo Fuchert, Dieu Vo, Rita Groß-Hardt

**Affiliations:** 1 University of Bremen, Centre for Biomolecular Interactions, Leobener Straße 5, 28359 Bremen, Germany; 2 Max-Planck-Institute for Plasma Physics, Wendelsteinstraße 1, 17491 Greifswald, Germany

## Abstract

It is considered an inviolable principle that sexually reproducing organisms have no more than two parents and fertilization of an egg by multiple sperm (polyspermy) is lethal in many eukaryotes. In flowering plants polyspermy has remained a hypothetical concept, due to the lack of tools to unambiguously identify and trace this event. We established a high-throughput polyspermy detection assay, which uncovered that supernumerary sperm fusion does occur in planta and can generate viable polyploid offspring. Moreover, polyspermy can give rise to seedlings with one mother and two fathers, challenging the bi-organismal concept of parentage. The polyspermy derived triploids are taller and produce bigger organs than plants resulting from a regular monospermic fertilization. In addition, we demonstrate the hybridization potential of polyspermy by instantly combining three different *Arabidopsis* accessions in one zygote. Our results provide direct evidence for polyspermy as a route towards polyploidy, which is considered a major plant speciation mechanism.

## Abstract (web summary)

The fertilization of an egg by more than one sperm is typically lethal. Here, via a novel reporter assay, Nakel et al. report the generation of triparental triploid *Arabidopsis* plants, implying that polyspermy is a plausible route toward polyploidy during plant evolution.

## Introduction

The ultimate goal for the survival of all species on earth is to reproduce. This uncompromising principle has triggered the evolution of numerous adaptations. One strategy commonly employed by sexually reproducing eukaryotes is the production of tremendous amounts of sperm to maximize the likelihood of egg fertilization. High sperm to egg ratios, however, are associated with an increased risk of polyspermy. It has been suggested that polyspermy in animals is lethal due to genomic imbalance associated with an increase in ploidy and the mismanagement of centrosomes, which are absent in plants^1, 2–5^. Interestingly, some plant species tolerate additional genomic copies as demonstrated by interploidy crosses and electropulse-assisted in vitro multi-gamete fusion^6–9^. To reduce the risk of supernumerary sperm fusion, eukaryotes have evolved polyspermy barriers, which are implemented at different levels in the reproductive process^10^. A common mechanism found in animals and plants is an egg cell block, which is mounted after gamete fusion and imposes chemical or physical barriers to reduce the risk of further sperm entry^11–15^.

As a characteristic feature of flowering plants, a pollen tube delivers two sperm cells that are essential for the fertilization of the two female gametes, the egg and the central cell. Upon successful gamete fusion, a pollen tube block is mounted, which typically prevents female gametes from being challenged with additional sperm cells^16–17^. However, *Arabidopsis* ovules are infrequently targeted by two pollen tubes^17–19^, which could create a chance for polyspermy to take place.

Naturally occuring plant polyspermy is hardly investigated; there are a couple of reports suggesting a polyploid with potentially polyspermic origin^20, 21^. However, the main route towards polyploids is considered to be unreduced gametes^22^ and this route could not be ruled out in these reports. In the work of Vigfússon (1970), the term polyspermy is used while actually referring to polytubey, the attraction of supernumerary pollen tubes^23^. In 1947 Hagerup reported on two sperm cells in orchids, which he interpreted to be both in the process of fusion with one egg cell^24^. However, the developmental consequences of this single observation are not clear. Thus, the concept of naturally occuring polyspermy in higher plants has remained hypothetical^15^.

We here report on a high-throughput polyspermy detection tool that employs the GAL4/UAS two-component system. This assay enables us to trace polyspermy in planta. Our results provide evidence that polyspermy is a route towards sexual polyploidization and challenge the biparental mode of sexual reproduction. Furthermore, we directly combined the genomes of three different *Arabidopsis* accessions, demonstrating the hybridization potential of polyspermy.

## Results

### Establishment of an in planta polyspermy detection tool

**Fig. 1:**
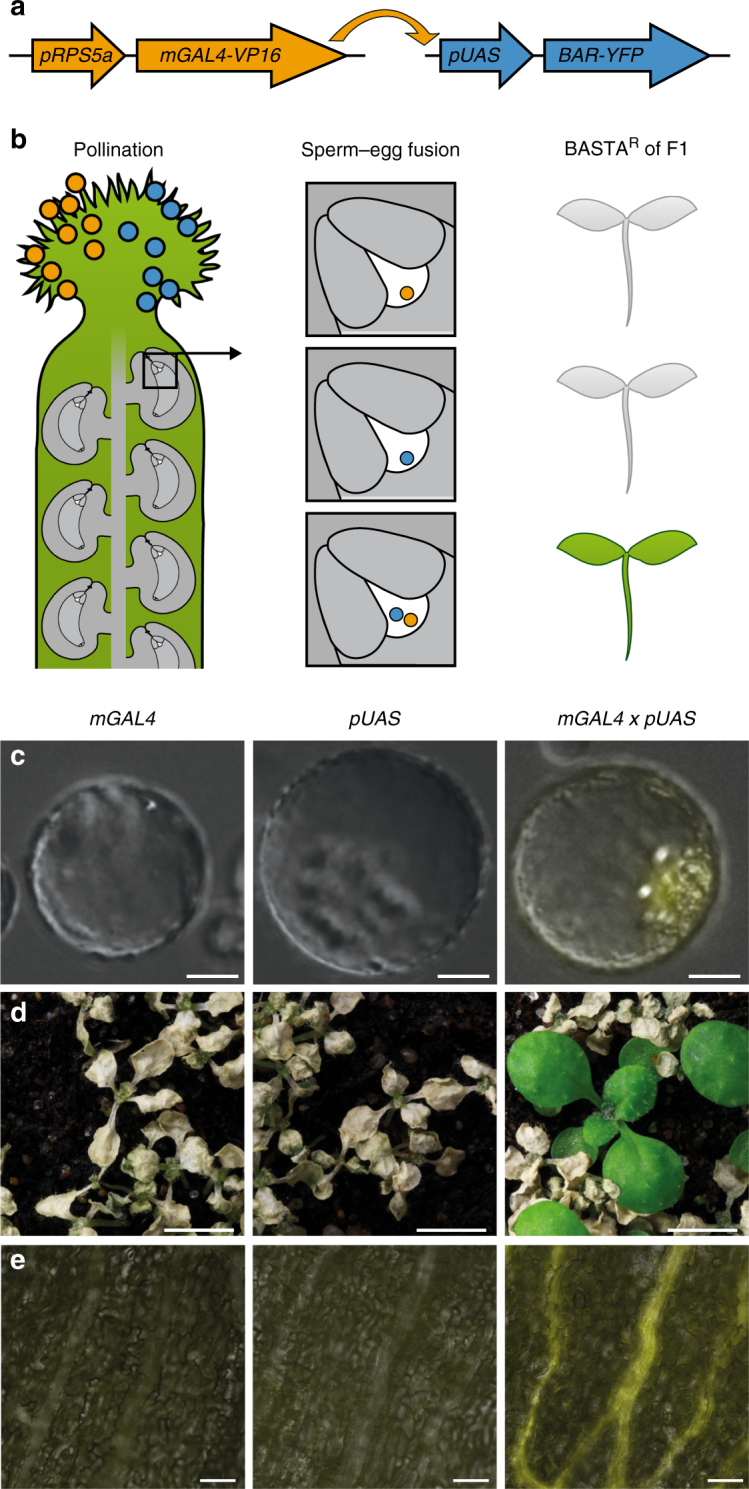
Establishment of a high-throughput polyspermy detection assay. **a** HIPOD comprises the synthetic transcription factor *mGAL4* driven by the ubiquitous *RPS5a* promoter, which transactivates the *UAS* promoter driving a herbicide resistance-conferring *YFP*-tagged *BAR* gene. **b** Pollen from PD1 (orange) and PD2 (blue) harboring the individual components of the two-component system are applied to the stigma of a gynoecium (green). Fusion of two sperm from two different fathers (orange, blue) to the egg cell (white) but not monospermy enables transactivation resulting in herbicide-resistant F1 plants. **c** Fluorescence microscopy of protoplasts transformed with *pRPS5a::mGAL4-VP16* (left), *pUAS::BAR-YFP* (middle) or cotransfected with *pRPS5a::mGAL4-VP16* and *pUAS::BAR-YFP* (right). **d** Herbicide-treated F1 plants resulting from selfing of *pRPS5a::mGAL4-VP16/* + (left), selfing of *pUAS::BAR-YFP/* + (middle), and a cross between *pRPS5a::mGAL4-VP16/-* and *pUAS::BAR-YFP/-* (right). **e** YFP fluorescence analysis in sepals of F1 plants resulting from selfing of *pRPS5a::mGAL4-VP16/* + (left), selfing of *pUAS::BAR-YFP/* + (middle), and a cross between *pRPS5a::mGAL4-VP16/-* and *pUAS::BAR-YFP/-* (right). Scale bars, 15 µm **c**, 0.2 cm **d**, and 50 µm **e**

In order to tackle the technical limitations of detecting the presumably rare event of polyspermy, we established a high-throughput polypaternal breeding design, which we termed HIPOD. HIPOD discriminates against seedlings of monospermic, i.e., unipaternal origin and positively selects seedlings of bipaternal origin, which can only arise from polyspermy. The assay consists of two pollen donors, which harbor the individual elements of the two-component system, *mGAL4-VP16/pUAS*^25^. Pollen donor 1 (PD1) contains the heterologous transcription factor *mGAL4-VP16* under the control of the ubiquitous *RPS5a* promoter (Fig. 1a), while PD2 contains an herbicide resistance-conferring *BAR* gene tagged to a yellow fluorescent protein (*BAR-YFP*). This latter construct is driven by the mGAL4-responsive *UAS* promoter. Seedlings resulting from monospermy will be rendered sensitive to the herbicide BASTA and die upon herbicide treatment. By contrast, combinations of both constructs, which can only result from polyspermy, will give rise to herbicide-resistant progeny (Fig. 1b). Please note that polyspermy events involving two sperm from either only PD1 or only PD2 do not deliver the two components necessary for herbicide resistance and hence escape detection. These monopaternal polyspermy categories are expected to account for 50% of all polyspermy events provided that there is no fertilization bias in favor of either PD1 or PD2. To address this issue we determined the segregation of *pUAS* and *GAL4* in F1 plants not subjected to the herbicide and found no substantial bias towards any of the constructs (*pUAS:GAL4* ratio of 248:258).

We first tested the HIPOD system in a transient expression assay using *Arabidopsis* protoplasts. Transformation with only one of the constructs yielded YFP-negative protoplasts. By contrast, protoplasts that have been cotransfected with both HIPOD constructs exhibited clear YFP fluorescence (Fig. 1c). We next transformed the individual constructs into distinct plants, thereby generating PD1 and PD2. While the propagation of either PD1 or PD2 resulted in herbicide sensitive plants, reciprocal crosses between PD1 and PD2 yielded herbicide-resistant and YFP positive progeny (Fig. 1d, e).

### HIPOD identifies plants with three parents

**Fig. 2:**
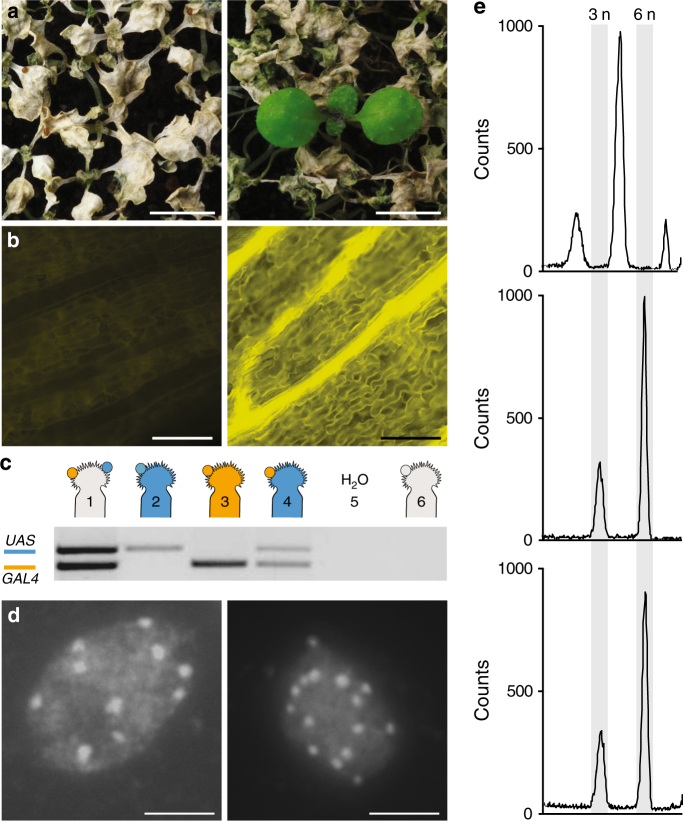
HIPOD identifies plants with three parents. **a** Herbicide-treated offspring of diploid wild type (left) and plants recovered from HIPOD (right). **b** YFP fluorescence analysis of diploid wild type (left) and plants recovered from HIPOD (right). **c** Multiplex PCR targeting *pUAS::BAR-YFP* (blue) and *pRPS5a::mGAL4-VP16* (orange) in (1) herbicide-resistant plant recovered from HIPOD, (2) *pUAS::BAR-YFP/* + , (3) *pRPS5a::mGAL4-VP16/* + , (4) *pUAS::BAR-YFP/−*, *pRPS5a::mGAL4-VP16/−*, (5) water control, (6) wild-type control. The crossing scheme resulting in the F1 plants analyzed is indicated in the cartoon. **d** DAPI stained chromosome spreads of a diploid wild-type plant (left) and a plant recovered from HIPOD (right). **e** Flow cytometric analysis of diploid wild-type plant (upper panel), triploid wild-type plant (middle panel) and a herbicide-resistant plant recovered from HIPOD (lower panel). Scale bars, 0.4 cm **a**, 100 µm **b**, and 5 µm **d**

To implement HIPOD as a polyspermy detection tool, we next provided PD1 and PD2 as distinct pollen donors onto wild-type flowers. We processed a total of 120644 seeds, as determined by a newly established segmentation-based object recognition software (Supplementary Fig. 1). Following herbicide treatment of the F1 generation, we detected 7 herbicide-resistant seedlings (Fig. 2a). Upon inspection of these plants under the fluorescence microscope, we detected YFP fluorescence in these plants (Fig. 2b and Supplementary Fig. 2a, b), indicating successful activation of the *YFP* gene present in PD2. To determine whether these plants are indeed of triparental origin, we carried out a multiplex PCR using DNA isolated from each plant and investigated the presence of both the PD1 and PD2 constructs. The result shows that all plants segregated both HIPOD constructs (Fig. 2c, Supplementary Figs. 2c and 5), indicating that the genetic material of two different fathers had been transmitted to a single egg cell. The introgression of more than one paternal copy implies that the resulting plants exhibit an increased ploidy. Notably, flow cytometry of all seven plants revealed a profile characteristic to triploid plants indicating that the nuclear genomes of two pollen donors had been inherited by a single egg cell (Fig. 2e and Supplementary Fig. 2d). To substantiate this result, we performed a complementary approach and determined the chromosome number of the herbicide-resistant plants by chromosome spreads. *Arabidopsis thaliana* contains five different chromosomes and in the diploid Landsberg *erecta* accession used in this study, they exist in two copies. Notably, in nuclei of the herbicide-resistant offspring recovered from HIPOD, we detected 15 instead of 10 chromosomes (Fig. 2d), confirming the triploid nature of these plants.

Our results establish HIPOD as a powerful tool to detect polyspermy, and demonstrate for the first time that flowering plants can give rise to polyspermy-induced viable seedlings with three instead of two parents: one mother and two fathers. It remains an open question, whether egg cell polyspermy is associated with central cell polyspermy. This is an interesting topic for future research as the central cell has been shown to be sensitive to supernumerary paternal copies^26^.

### Triparental triploids are taller and produce bigger organs

**Fig. 3:**
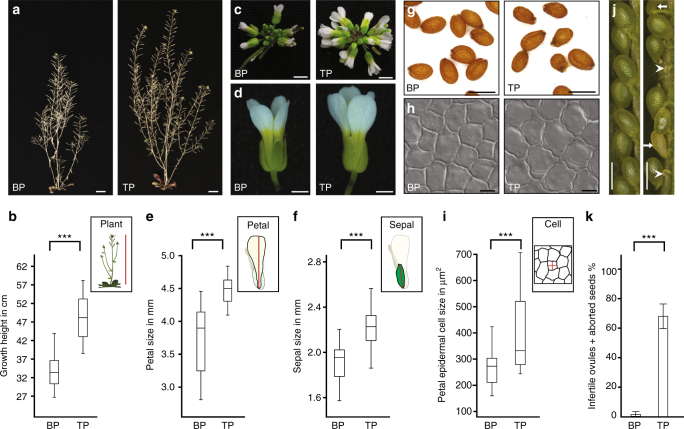
Polyspermy-induced triploid plants are taller and produce bigger organs than plants originating from a regular fertilization mode. **a**, **b** Growth height comparison between biparental diploid plants (BP; *n* = 19), and herbicide-resistant triploid triparental plants (TP; *n* = 7). **c**, **d** Inflorescence and flower of BP and TP plants. **e** Petal size of BP (*n* = 68) and TP plants (*n* = 104). **f** Sepal size of BP (*n* = 89) and TP plants (*n* = 107). **g** Seed morphology of BP and TP plants. **h**, **i** Size of petal epidermis cells in BP (*n* = 58) and TP plants (*n* = 93). **j**, **k** Silique analysis of 2n BP (*n* = 19) and 3n TP (*n* = 39) plants. Siliques are showing dark green seeds, sterile ovules (white arrowhead) and abnormal seeds (white arrow). **k** Graph shows mean ± s.d. P values (***p < 0.001) report significance by t-test. Box plots show median, quartiles, maximum and minimum. Scale bars, 2 cm **a**, 2 mm **c**, 1 mm **d**, 0.5 mm **g**, 5 µm **h**, 0.5 mm **j**

We next characterized the growth and viability of these genuine triparental plants in comparison to control plants, originating from a regular monospermic fertilization mode, and assessed various life-history traits. In comparison to control plants, overall plant height of triparental plants was increased by a factor of 1.5 and the plants gave rise to bigger inflorescences and flowers (Figs 3a–d). In addition, we found that triparental plants produced significantly larger organs when compared to control plants: Petal and sepal size were increased by 20.7% and 17.0%, respectively (Fig. 3e, f). To determine whether organ hypertrophy was caused by cell proliferation or expansion, we determined the size of petal epidermis cells. The result shows that cell size instead of number is substantially increased in the triploid plants (Fig. 3h, i), corroborating previous findings that have suggested a positive correlation between DNA content and cell size in various organisms, including *Arabidopsis*, *Drosophila*, and zebrafish^27^. As shown for example in *Arabidopsis*, meiosis in triploid plants results in aneuploidy; an imbalance in chromosome number^28^. In fact, we observed that fertility in triparental plants is substantially reduced accompanied by the segregation of shriveled and malformed ovules and seeds, which is compatible with aneuploidy associated defects (Fig. 3g, j, k). The effects observed on growth and fertility are similar to reports about triploid plants, which were manually generated by crossing diploid and tetraploid parents^28^.

Our results provide direct evidence for polyspermy as a route towards polyploidy, which is considered a major plant speciation mechanism^29, 30^.

### Polyspermy-induced hybridization of three accessions

**Fig. 4:**
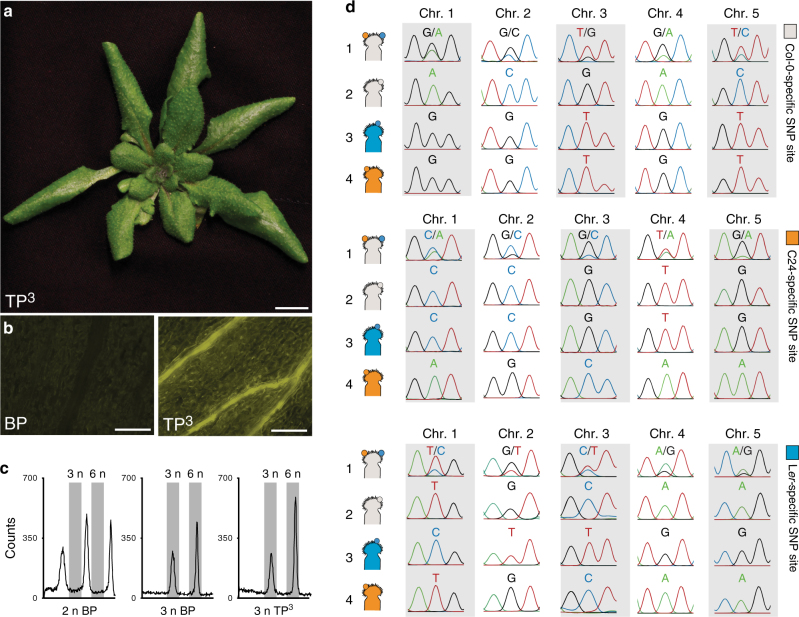
Polyspermy-induced hybridization of three accessions. **a** Herbicide-resistant plant recovered from three accession HIPOD cross between Col-0, C24 *pRPS5a::mGAL4-VP16/ + *and L*er pUAS::BAR-YFP/ + *(TP^3^). **b** YFP fluorescence analysis of a diploid biparental plant (BP) and a triploid triparental plant recovered from a three accession cross (TP^3^). **c** Flow cytometry analysis of 2n biparental (2n BP), 3n biparental (3n BP) and 3n triparental plant recovered from a three accession cross(3n TP^3^). **d** Analysis of accession-characteristic RFLPs on different chromosomes (Chr.) in TP^3^ (1), Col-0 (gray) (2), Ler (blue) (3), and C24 (orange) (4). The crossing scheme resulting in the F1 plants analyzed is indicated in the cartoon. Scale bars, 1 cm **a**, 100 µm **b**

The polyspermy-induced route towards polyploidization has further important evolutionary implications, as it bears the potential to instantly hybridize three different germplasms. To test this hypothesis, we aimed to combine three genetically distinct *Arabidopsis* accessions in a three-parent cross. In order to facilitate the detection of triparental offspring, we introduced the *pUAS* HIPOD responder construct into the *Arabidopsis* L*er* accession and the *GAL4* HIPOD driver construct into the C24 accession. Following pollination of a third accession, Col-0, with pollen from the two distinct fathers we were, indeed, able to recover two viable herbicide-resistant plants (TP^3^ 1 and 2, Fig. 4a), which exhibit delayed flowering compared to the parental accessions (Supplementary Fig. 3). TP^3^ 1 and 2 passed the previously introduced tests for triparental plants: They segregated an active YFP, and exhibited a triploid profile in the flow cytometry assay (Fig. 4b, c). To test whether, in fact, the chromosomes of three different accessions had been transmitted, we targeted accession specific restriction fragment length polymorphisms (RFLPs) on each of the five chromosomes. Notably, we detected polymorphisms characteristic for each of the three accessions on all chromosomes (Fig. 4d, Supplementary Fig. 4), indicating that all three nuclear genomes have been inherited. To our knowledge this is the first direct evidence for an instant hybridization of three different germplasms in one zygote.

## Discussion

Polyploidization is considered as a major speciation mechanism^29, 30^, mainly due to the instant reproductive isolation associated with additional genomic copies. It is estimated that up to 70% of flowering plant species are polyploid^31^. Up to now, it was generally assumed that polyploidization arises from one of the two scenarios: (i) Unreduced gamete formation due to rare meiotic or mitotic catastrophes during gametogenesis^32, 33^ or (ii) Somatic doubling that occur in the sporophytic tissue of the plant^22, 34^. The HIPOD assay for the first time allows tracing the rare, but evolutionary highly relevant event of polyploidization. Our results provide tangible evidence for polyspermy as a natural source of plant polyploidization. But how significant is this scenario? As an approximation, we calculated the frequency of polyspermy-induced polyploid plants on the basis of the number of triparental plants recovered (7) and the number of seeds processed (120644). In addition, we took into account that HIPOD at its best detects 50% of all polyspermy events as supernumerary sperm contributions involving sperm from a single father escape detection.

On the basis of this conservative calculation, we obtained polyspermy-induced polyploids at a frequency of 0.012%. When extrapolating this rate to the approximately 45000 seeds generated on average by *Arabidopsis* under the growth conditions used in this study (Supplementary Table 1), our data implies that every plant can give rise to about 5 polyspermy-induced polyploid plants. In comparison, unreduced gamete formation in predominantly selfing Brassicaceae species has been reported to occur on average at a frequency of 1.8%^35^. However, due to the lack of tools to trace the fate of unreduced gametes it is not known how often this scenario leads to polyploid plants.

Our finding that polyspermy in flowering plants can give rise to plants that contain the nuclear genome of three instead of two parents can not only lead to a paradigm shift regarding our understanding of the mechanisms underlying polyploidization, but also expands the current bi-organismal concept of parentage. In addition, it will open up new horizons for plant breeding and eukaryotic polyspermy research.

## Methods

### Growth conditions

Plants were germinated on soil in a Conviron MTPS growth chamber under long day conditions (16 h light/8 h dark) at 23 °C until bolting. Afterwards they were transferred to a Conviron MTPS growth chamber under long day conditions at 18 °C.

### HIPOD proceedings

For the HIPOD assay 3-8 closed flower buds per inflorescence were emasculated. Pollination was performed two to three days later with pollen grains harvested separately from plants harboring *pRPS5a:mGAL4-VP16/ + *and *pUAS::BAR-YFP/ + *. Pollen was collected using a vacuum based device adopted from José Feijó lab^36^ and pollen was applied on the stigmatic surface with two brushes. In three independent experiments a total of 120644 seeds were processed. For the three accession HIPOD cross we processed 9493 seeds in two independent experiments.

### Establishment of a seed count software

Seeds were placed on a 10 × 15 cm sheet and images were captured using a Canon EOS 700D camera with a Canon-EF-100mm-f-2-8-Macro-USM objective lens set to manual mode (ISO100; f/2.8; 1/8 s; Flash on). Count_seeds.py is a computer code written in Python to count plant seeds in three channel color images. Usage of count_seeds.py requires a standard installation of Python, together with the extension library OpenCV^37^. In the first step, the image regions displaying seed material are determined. The seeds occupy a specific volume in the 3D color space. In order to deal with color and intensity changes of the illumination, a white reference is calculated for a specified number of subframes and the color space is normalized to this white reference. To minimize color noise, regions of comparable color are reduced by bilateral filtering. The color volume representing seed material has been determined by hand before the first run of the routine and has to be adapted if the illumination setup changes drastically. The image regions displaying color values in the color volume of the seeds are copied to a binary image, where pixels displaying seed material have a value of one and all other pixels of zero. To detect seeds of different sizes (Supplementary Fig. 1a) and separate individual seeds in a cluster (Supplementary Fig. 1b), a two-step approach is used: In the first step, connected regions in the binary image with an area smaller than a predefined threshold value are definitely single seeds, which are labeled directly and removed from the binary image. In the second step the distance transform of the binary image of remaining seeds is calculated. For seed separation in the remaining regions a template pattern is calculated as the distance transform of a circle with a characteristic radius (adjusted manually before the first run). The distance transform of the seed image is correlated with this template pattern and the local maxima of the resulting 2D correlation function are detected by a local extremum filter. These detected maxima are single seeds. Finally, all seeds are represented by the center of mass of their connected structures of maximum correlation and these centers of mass are labeled. The number of unique labels is the number of seeds. The detection of residual plant material achieved by the initial color volume analysis is displayed in Supplementary Fig. 1c. The requirement to automatically detect seed clusters and residual plant material introduces a source of detection errors. Therefore, three series of independent measurements were performed. The seeds were spread and photographed ten times independently (Supplementary Fig. 1d, e). On the basis of data reproducibility indicated in Supplementary Fig. 1e, we put a threshold at 6000 seeds/image. Within this range, we compared software to manual counts yielding a difference of ± 1.5%.

### Plant phenotyping

Growth height, sepal length, petal length and cell size were measured from digital images. Dissected sepals and petals were mounted in 70% alcohol to measure proximodistal dimensions. For cell size measurements petals were cleared in chloral hydrate:glycerin:ddH_2_O (8w:2 v:1 v) and average cell sizes were calculated at the adaxial side, from the number of cells per unit area of digital micrographs^38^. All size measurements were calculated using Image J software. The fertility assay was carried out on 3-4 week old siliques which were opened with a syringe (BD Micro-Fine + ) and ovules were inspected using a Leica S6E or S8apo stereomicroscope (Leica, Germany). The total number of seeds produced per wild-type plant was assessed by collecting all mature siliques. Digital images of the respective seeds were processed using count_seeds.py.

### Microscopy

Images were taken using Leica DMI6000b epifluorescence inverted microscope, equipped with a YFP ET, k (material number 11504165) and a DAPI ET, k (material number 11504203) filter cube. For *YFP* expression analysis, dissected cotyledons or sepals from opened flowers were transferred to 10% glycerol and data were collected. For chromosome spread, data from DAPI stained samples were collected with the aid of 100X oil objective and an immersion oil Immersol 518F (Zeiss, Germany).

### Ploidy analysis

Ploidy analysis was performed employing two different methods: flow cytometry and chromosome spread. For flow cytometry, one or two leaves were harvested and chopped using a razor blade in a petri dish with nuclei extraction buffer (Partec CyStain). Staining reagent (Partec CyStain UV Precise-Kit) was added and specimens were incubated at RT for 1 min. The liquid was passed through a 50 µM nylon mesh and samples were analyzed using Partec CyFlow ploidy analyzer.

Chromosome spreads were carried out from representative plants of each genotype according to published protocols^39^. Flower buds were harvested and pretreated with a solution containing 2.5 mM 8-hydroxyquinoline, 100 µM oryzalin, and 100 µM colchicine for 4 h, followed by incubation in Carnoy’s fixative solution (absolute ethanol:chloroform:glacial acetic acid, 6 v:3 v:1 v) overnight. Prior to enzyme treatment, sepals and petals were removed and samples were washed twice with 10 mM citrate buffer pH 4.5 (buffer stock 0.1 M citric acid: 0.1 M sodium citrate, diluted 1:10). Subsequently samples were incubated in enzyme solution (0.3% w/v Cytohelicase, 0.3% w/v pectolyase, and 0.3% w/v cellulase in citrate buffer) at 37 °C for 1 h. The enzymatic reaction was stopped by treatment with ice cold citrate buffer. Samples were transferred onto glass slides and incubated in 5 µl of 60% acetic acid until the solution was completely evaporated. Staining of chromosomes was then carried out by applying DAPI (1 µg/ml) in Vectashield antifade mounting medium. Unless otherwise stated all steps were performed at room temperature. Samples were analyzed with a Leica DMI6000b epifluorescence inverted microscope equipped with DAPI filter, 100× oil objective and with immersion oil (Immersol 243 518F, Zeiss, Germany).

### Determining frequencies of polyspermy-induced polyploids

As an approximation, the frequency of polyspermy-induced polyploid plants was calculated on the basis of total seed counts and the number of detectable triparental seedlings, as evidenced by BASTA resistance and positive identification of both paternal HIPOD constructs. In addition, it was taken into account that HIPOD only detects 50% of all polyspermy events: Frequency of polyspermy-induced polyploidization equals (2× # TP/ # seeds)×100.

### Cloning strategy

The HIPOD constructs were assembled on the basis of pGIIHyg and contain the *pUAS/GAL4* transactivation system kindly provided by Dolf Weijers. The *pUAS* sequence was amplified using 5′-ATGGCGCGCCGCATGCCTGCAGGTCGGA-3′ and 5′-ATTTAATTAACGGGGATCCGGTTCTCTC-3′. Subsequently *YFP* was cloned into a pGIIHyg Vector using NotI/SacI restriction sites. The *BAR* gene was cloned from pGIIBar using 5′-ATTTAATTAAATGAGCCCAGAACGACGCCC-3′ and 5′-ATGCGGCCGCGATTTCGGTGACGGGCAGGAC-3′ and integrated into the vector using PacI/NotI. In a final step *pUAS* was inserted upstream using AscI/PacI. *GAL4* was amplified by using 5′-ATTTAATTAAATGAAGCTCCTGTCCTCCATCGA-3′ and 5′-ATGCGGCCGCCTACCCACCGTACTCGTCAATTC-3′. In addition, the pGIIHyg vector backbone was used and *GAL4* was inserted using PacI/NotI restriction sites. Subsequently, *pRPS5a* was inserted upstream of *mGAL4-VP16* using AscI/PacI. Further cloning details upon request.

### Plant transformation

*Arabidopsis thaliana* (L*er + *C24) was transformed with the binary vector constructs (*pRPS5a::mGAL4-VP16* and *pUAS::BAR-YFP*) by *Agrobacterium tumefaciens* assisted floral dipping^40^.

### PCR-Based genotyping

Plants were genotyped by multiplex PCR using primers 5′-TATAGGGCGAATTGGGTACC-3′ and 5′-GGAACTGGCATGACGTGGGTTT-3′ for *pUAS::BAR-YFP* and 5′-TCGTTTTCTCTGCCGTCTCTCT-3′ and 5′*-*CCCTTGTTGCTGCTCTCCTC-3′ for *pRPS5a::mGAL4-VP16*.

### RFLP Analysis

Primers flanking Col-0, C24, and L*er* characteristic RFLPs on all five chromosomes were designed. RFLPs were chosen on the basis of the POLYMORPH web tool^41, 42^ (Supplementary Table 2).

### Statistical analysis

Unless otherwise stated experiments were repeated three times independently and statistical analysis was performed using two-tailed t-test: *p < 0.05; **p < 0.01; ***p < 0.001. Figure 3b plant height compares a total of 19 diploid wild type (*n* = 19) to all 7 triparental plants generated in this study (*n* = 7). Figure 3e: Petal size is based on 68 wild-type petals (*n* = 68) and 104 triparental petals (*n* = 104). Figure 3f: Sepal size is based on 89 wild-type sepals (*n* = 89) and 107 triparental sepals (*n* = 107). Figure 3i: Petal epidermal cell size was determined from 7 triparental plants using a total of 93 petals and 10 wild-type plants using a total of 58 petals. Figure 3k: Silique analysis was performed twice and the experiment was conducted blind. In total 19 wild-type siliques (*n* = 19) corresponding to 516 seeds and 39 siliques of triparental plants (*n* = 39) corresponding to 1016 seeds were counted.

### Data availability

All data generated or analyzed during this study are included in this published article (and its Supplementary Information Files) or are available from the corresponding author upon request. The computer code generated during the current study is available from the corresponding author on request.

## Electronic supplementary material

Supplementary Info
