# Publisher Correction: Triparental plants provide direct evidence for polyspermy induced polyploidy

**DOI:** 10.1038/s41467-018-03823-7

**Published:** 2018-04-11

**Authors:** Thomas Nakel, Dawit G. Tekleyohans, Yanbo Mao, Golo Fuchert, Dieu Vo, Rita Groß-Hardt

**Affiliations:** 1 University of Bremen, Centre for Biomolecular Interactions, Leobener Straße 5, 28359 Bremen, Germany; 2 Max-Planck-Institute for Plasma Physics, Wendelsteinstraße 1, 17491 Greifswald, Germany

Correction to: *Nature Communications* 10.1038/s41467-017-01044-y; published online 18 October 2017.

This Article contained errors in Fig. 3 that were brought to our attention by the authors during the production process but, inadvertently, were not corrected before publication. The tick marks on the *y*-axis in panels b, f, and k, and the median line in the box-and-whisker plot for biparental diploid plants (BP) in panel i were shifted downwards by up to 2 mm. This has now been corrected in both the PDF and HTML versions of the Article.

