# Supplementary material for: Triparental plants provide direct evidence for polyspermy induced polyploidy
Source: Nat Commun. 2017 Oct 18;8:1033. doi: 10.1038/s41467-017-01044-y (PMC5647324; doi:10.1038/s41467-017-01044-y)
Supplement: Supplementary file 1 — Supplementary Info [file 41467_2017_1044_MOESM1_ESM.pdf]

a Sensitivity to different object sizes

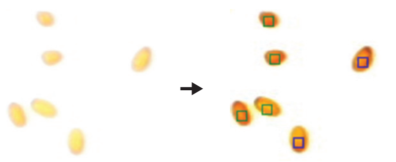

b Recognition of individual seeds in seed clusters

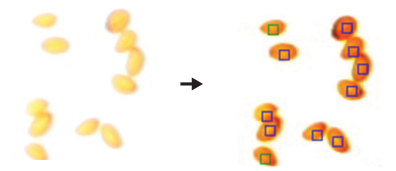

c Discrimination of residual plant material

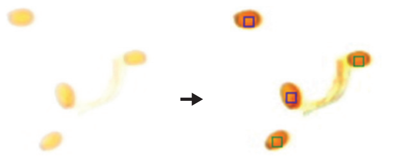

d

S1	S2	S3	S4
867	2661	6343	8550
867	2719	6479	9619
861	2750	6353	8016
864	2726	6488	9245
862	2755	6438	8887
860	2668	6521	8715
867	2696	6586	8981
869	2702	6561	9536
867	2673	6493	9760
859	2689	6393	9505

e

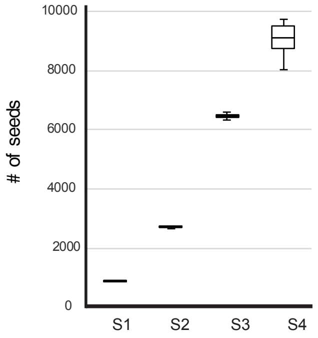

Supplementary Fig. 1: Seed counting using segmentation based object recognition. (**a-c**) Count_seeds.py software (a) recognizes seeds of different size, (b) identifies individual seeds in seed clusters, and (c) discriminates against residual plant material. Squares indicate objects identified as seeds after the first (green) and the second step (blue). (**d** and **e**) Depicted are scores of four seed samples (S1-S4), which were spread and photographed ten times independently and computed using count_seeds.py software.

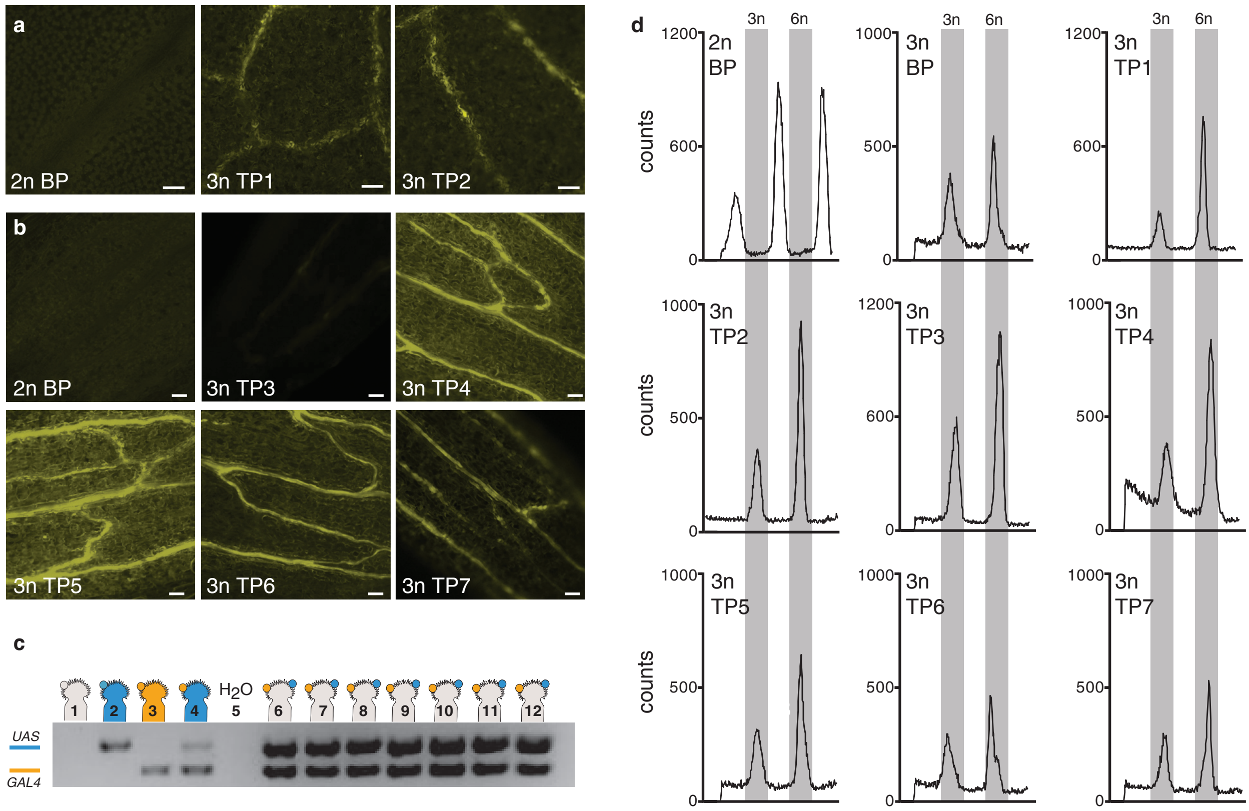

Supplementary Fig. 2: HIPOD identifies triparental plants. (**a**) YFP fluorescence analysis in cotyledons of 2n biparental wild-type (2n BP) and triparental plants recovered from HIPOD (TP1 and TP2). (**b**) YFP fluorescence analysis in sepals of 2n biparental wild-type (2n BP) and triparental plants recovered from HIPOD (TP3-TP7). (**c**) Multiplex PCR targeting *pRPS5a::mGAL4-VP16* (orange) and *pUAS::BAR-YFP* (blue), in wild type (1), PD2 *(pUAS::BAR-YFP/+)* (2) , PD1 *(pRPS5a::mGAL4-VP16/+)* (3), cross between PD1 *X* PD2 (4), water control (5), and all seven herbicide-resistant plants recovered from HIPOD (6-12). The crossing scheme resulting in the F1 plants analyzed is indicated in the cartoon. (**d**) Flow cytometric analysis of diploid wild-type bipaternal plant (2n BP), triploid wild-type bipaternal plant (3n BP) and all seven herbicide-resistant triparental plants recovered from HIPOD (3n TP1-7). Scale bars, 100 µm (a), 50 µm (b).

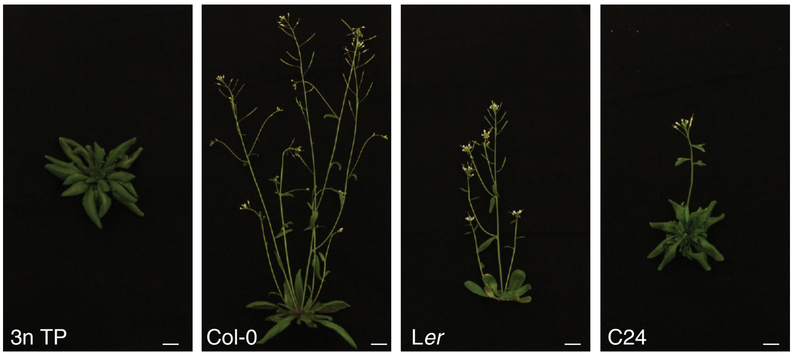

Supplementary Fig. 3: Polyspermy-induced hybrids combining three accessions exhibit delayed flowering. HIPOD- recovered triparental plant (3n TP) and the respective diploid parental accessions, Col-0, L*er*, and C24 41 days after sowing. Scale bar, 2cm.

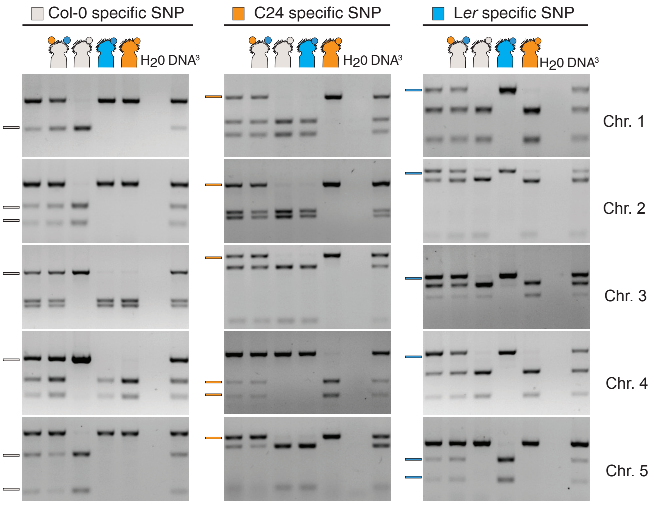

Supplementary Fig. 4: Herbicide resistant seedlings from a cross of Col-0, C24 and L*er* segregate restriction fragment length polymorphisms (RFLPs) specific for all three accessions. Col-0 (grey), C24 *pRPS5a::mGAL4-VP16/+ (orange)* and L*er* *pUAS::BAR-YFP/+* (blue) were crossed using the scheme as indicated in the cartoons. All five chromosomes of the resulting F1 were analyzed with respect to RFLPs characteristic for Col-0 (grey bar), C24 (orange bar), and L*er* (blue bar). The two F1 plants resulting from the three accession cross (boxed) have been recovered following herbicide treatment. H_2O and a mixture of DNA from all three accessions (DNA^3) represent negative and positive controls, respectively.

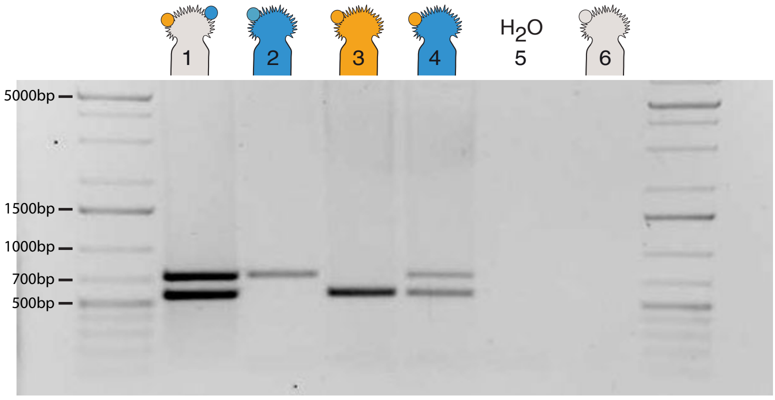

Supplementary Figure 5: Multiplex PCR targeting *pUAS::BAR_YFP* (blue) and *pRPS5a::mGAL4-VP16* (orange) in (1) herbicide-resistant plant recovered from HIPOD, (2) *pUAS::BAR-YFP/+*, (3) *pRPS5a::mGAL4-VP16/+*, (4) *pUAS::BAR-YFP/-*, *pRPS5a::mGAL4-VP16/-*, (5) water control, (6) wild-type control. The crossing scheme resulting in the F1 plants analyzed is indicated in the cartoon. Molecular size marker (Gene ruler™ 1kb Plus DNA Ladder).

Plant ID	Total seed count	Shattered siliques	Total seed count, extrapolation
1	44219	75	48119
2	41525	42	43709
3	36143	89	40771
4	34531	110	40251
5	34684	92	39468
6	37830	10	38350
7	43124	68	46660
8	40647	97	45691
9	38630	100	43830
10	48306	53	51062
11	49704	49	52252
12	40961	94	45849
13	43443	66	46875
14	45146	70	48786
15	45203	43	47439
16	41133	67	44617
17	48239	73	52035
18	30771	18	31707
19	44665	71	48357
20	39291	39	41319
21	44390	49	46938
22	41892	70	45532
Average	41567	66	44983

Supplementary Table 1: Total seed count per plant. Seeds of 22 individual plants were scored. Shattered siliques which had lost their seeds could not be analyzed and are indicated. As approximation for total seed number, we included these objects by assigning them the score 51.8, which is the average seed number produced by one *Arabidopsis* silique, yielding the category “total seed count, extrapolation”.

Primer Name	Sequence of Primers 5'-3'	Restriction enzyme	Chromosome
DT51_Fw	CAGGACATCCACCTAGAAACAAGAAGAC	AluI	Chr. 1
DT52_Rev	TGAATGTGTGCAGATCATGGAGAAGGAC		
DT53_Fw	TGTACCTGGATTCGATAGAACTGC	NdeI	
DT54_Rev	AGAAGTCCAAGCATGTCCTATGGT		
DT55_Fw	TAAGATTGCTCAGAAGCTGAGTGAG	SacI	
DT56_Rev	CGACACACAATGTATCTGTTTACCC		
DT92_Fw	ACCTCTAAACGTGATTGCAACTGTGAG	HindIII	Chr. 2
DT93_Rev	TGTGCCAACAATCTCTCATGTACATGG		
DT59_Fw	TGGTCGTTGCTTAAGTTGTTAAGCTCG	PstI	
DT60_Rev	CAATACAAGACACTTTCTCCAATATGGG		
DT61_Fw	TGGTTCACATGTACGCCAAGTGTTACC	BamHI	
DT89_Rev	CTTCCCTTCATTAACCATCCCTGTGTG		
DT116_Fw	TTATTTCCGACTTTGTCTTTGCCATCG	SacI	Chr. 3
DT117_Rev	TGCTAGATTATGCCTTTGATCACAAGC		
DT65_Fw	GCTGCAATGGTAAACAAGGAGGAAGAG	HindIII	
DT66_Rev	CGGGATCATAATGCCGATATTCTTCGG		
DT67_Fw	TGTCTTCAGCATATATGCCGATTGGAG	HindIII	
DT68_Rev	CAATCTCTTCAGGTGAGATAATGAGCG		
DT96_Fw	ACACGCAAGAGAGAATTGGATGATACC	ClaI	Chr. 4
DT97_Rev	AGCAGAATCTCAATCTCGATGGTTGTG		
DT71_Fw	TCTCTTGCTCTCTTCTCTCTCACACAG	EcoRV	
DT72_Rev	ACCTCTTGTGGAGTATCAAGCCAAGTG		
DT98_Fw	TACAGAGTCCAAGTCGTTGTCATGCTC	PstI	
DT99_Rev	TGCGTGACTGCTATATCCTAAAGGAGG		
DT77_Fw	CAACGAGAAGTTGGATTGTGAGGTG	SpeI	Chr. 5
DT78_Rev	GGATGAAGGAGCATATGGAGATAGC		
DT79_Fw	GTATTCACGACCATCGTAGCTGTCCAC	SmaI	
DT80_Rev	AACTCGCCTCTGAAGCTGGTCAATGAG		
DT81_Fw	AACAGTAATGGTCCCCATTCACGGTGG	HindIII	
DT82_Rev	CCAATTAGACCTTGAAGGAGCAGGACC		

Supplementary Table 2: Oligomers used for RFLP analysis. Primers and restriction enzymes used to detect RFLPs characteristic to Col-0, L*er*, and C24 on 5 different chromosomes.
